# Faecal sample storage without ethanol for up to 24 h followed by freezing performs better than storage with ethanol for shotgun metagenomic microbiome analysis in patients with inflammatory and non-inflammatory intestinal diseases and healthy controls

**DOI:** 10.1186/s13104-024-06999-y

**Published:** 2024-11-15

**Authors:** Ida Marie Bruun Grønbæk, Sarah Mollerup, Sofie Ingdam Halkjær, Sarah Juel Paulsen, Mette Pinholt, Henrik Westh, Andreas Munk Petersen

**Affiliations:** 1 Gastrounit, Medical Section, Copenhagen University Hospital – Amager and Hvidovre, Kettegård Alle 30, 2650 Hvidovre, Denmark; 2 Department of Clinical Microbiology, Copenhagen University Hospital – Amager and Hvidovre, Kettegård Alle 30, 2650 Hvidovre, Denmark; 3 Department of Clinical Medicine, University of Copenhagen, Copenhagen, Denmark

**Keywords:** Faecal sample, Sample storage, Preservation, Ethanol, Gut microbiome, Shotgun metagenomics

## Abstract

**Objective:**

The influence of different faecal collection methods on metagenomic analyses remains under discussion, and there is no general agreement on which collection method is preferable for gut microbiome research. We compared faecal samples collected in tubes without preservatives with those containing 10 mL of 96% ethanol for gut microbiome research when the timeframe from defecation to freezing at – 80 °C was up to 24 h. We aimed to compare the collection methods on faeces from participants with inflammatory and non-inflammatory gastrointestinal disorders and healthy controls to investigate the most suitable method when considering data yield, human fraction of sequencing reads, and ease of use. We also examined the faecal sample homogeneity.

**Results:**

Faeces collected in tubes without preservatives resulted in more sequencing reads compared to faeces collected in tubes with 96% ethanol and were also easier to handle. The human fraction of total reads in faeces collected in ethanol from participants with inflammatory bowel disease was higher than all other samples. DNA extraction and sequencing from two different locations in the same faecal sample gave similar results and showed sample homogeneity.

**Supplementary Information:**

The online version contains supplementary material available at 10.1186/s13104-024-06999-y.

## Introduction

Multiple association studies have linked the gut microbiome composition to human health and various diseases including the gastrointestinal (GI) disorders irritable bowel syndrome (IBS) and Crohn’s disease (CD) [1–4]. With the advancement of shotgun metagenomics sequencing, mapping of the gut microbiome has become more accessible [5]. However, data on how different faecal collection methods influence metagenomic data and analyses is ambiguous [1, 2].

When conducting clinical trials that explore the association between GI diseases and the human gut microbiome, knowing the most effective approach is crucial. Methodological research plays an important role in ensuring the reliability and accuracy of such studies [1, 6]. Furthermore, a standardised faecal sample collection method should be feasible in clinical practice [7, 8]. Faecal sample collection without any preservatives and rapidly freezing at -80°C is considered the gold standard [1, 6, 7]. However, in most human clinical trials, immediate freezing or analysis of a faecal sample is not possible, and thus a need for both preservation and long-term storage may be required [7].

Ethanol is widely used as a preservative of biomaterial because of its ability to fixate DNA by inhibiting DNA-degrading enzymes [9, 10] and its low cost. This study aimed to determine the optimal faecal collection method (no preservative versus ethanol) in clinical gut microbiome research involving participants with various GI conditions where samples are stored for up to 24 h before freezing at – 80 °C. The ease of use for sample collection at home and laboratory handling was considered. A secondary aim was to test the homogeneity of the faecal samples as the macroscopic composition of the samples can vary greatly.

We focused on patients with IBS and CD. Knowing that CD is an inflammatory bowel disease (IBD), we sought to test the collecting methods on patients with a high level of faecal calprotectin, a sensitive marker for GI inflammation, to investigate how the two methods affect the amount of human DNA reads.

## Methods

### Study participants

Four patients with IBS, four patients with CD with an abnormal level of faecal calprotectin (above 50 mg/kg), and four healthy volunteers delivered faecal samples for this study. All participants with IBS and CD were adult patients originally recruited for clinical research projects at Copenhagen University Hospital Hvidovre. A family of four, without any known GI diseases, volunteered to participate as healthy controls.

**Fig. 1:**
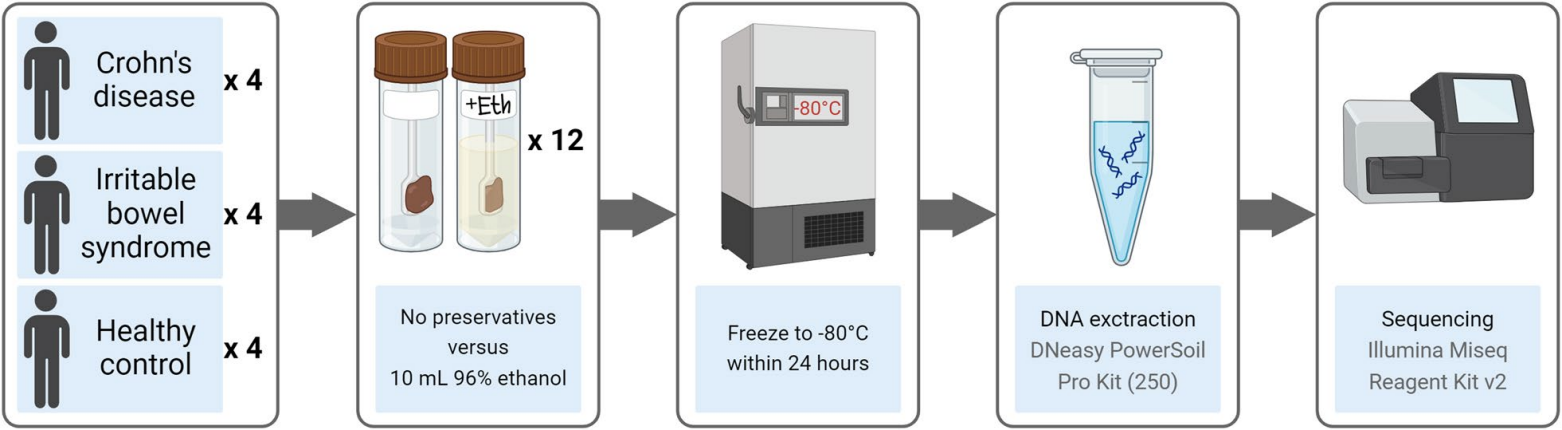
Illustration of the study setup. Faecal samples were continuously collected, frozen, and analysed

The study setup is illustrated in Fig. 1.

### Faecal specimen collection

Each study participant delivered a set of faecal samples consisting of one tube without preservatives and one tube added 10 mL of 96% ethanol. Both samples were collected from the same stool. The consistency of the samples varied from loose to solid. Participants were asked to collect the samples on the same day as they would be delivered to the research investigator to ensure that samples would be frozen to – 80 °C within 24 h. If participants knew that a toilet visit in the morning was not guaranteed, they were allowed to collect the faecal samples the evening before and store the samples in the refrigerator (5 °C) until departure from home. Regardless, the 24-h time interval from defecation to freezing was maintained.

### DNA extraction and sequencing

DNA extractions were performed manually using the DNeasy PowerSoil Pro Kit (250) (cat. nr. 47016, Qiagen, Hilden, Germany) following the manufacturer’s instructions. A 10 µL inoculation loop (cat. nr. 86.1562.959, Sarstedt, Nümbrecht, Germany) was used for sampling from the original non-homogenised stool sample. The sample was transferred to a tube containing beads, and lysis-buffer (CD1), and vortexed briefly to mix. Next, the tube was placed vertically in a styrofoam sample holder on a vortex mixer (Edison, New Jersey, USA) and mixed at maximal power for 10 min. Library preparation was performed automatically on a Biomek 4000 robot (Bechman Coulter, Indianapolis, USA) with 24 samples processed at a time. The library preparation was performed using the Nextera XT DNA Library Prep (cat. nr. FC-131-1096, Illumina, San Diego, California, USA) with bead-based normalisation and Nextera XT Index kit v2 (cat. nr. FC-131-2001/FC-131-2004, Illumina, San Diego, California, USA). The library preparation was performed as recommended by the kit manufacturer, except for an adjusted volume of AMPure XP beads during the final library purification following indexing (25 μL rather than 30 μL).

Sequencing of samples was performed on an Illumina MiSeq with Illumina MiSeq^®^ Reagent Kit v2 (300 Cycles) (cat.nr. 15,033,418, Illumina, San Diego, California, USA), generating 150 base pair paired-end reads.

### Test of sample homogeneity

The faecal samples from participants with CD were each DNA extracted and sequenced twice to investigate the homogeneity of the sample as well as the stability of the process. In the process of DNA extraction, samples were deliberately taken from two different locations in the stool. The procedure was performed by the same laboratory personnel each time. When evaluating data yield, only the first set of CD samples was included.

### Bioinformatic analysis

Raw FASTQ read files were trimmed using fastp v. 0.20.1 [11] with –qualified_quality_phred of 20 and minimum read length of 50. FastQC v. 0.11.8 [12] was used to evaluate the quality of the reads before and after trimming. Depletion of human sequences was performed by aligning the trimmed reads to the human genome (hg38, University of California, Santa Cruz) using bowtie2 v. 2.3.4.1 [13] with end-to-end alignment and maximum fragment length for valid paired-end alignments (-X) of 2000. Clade-based microbial profiling of the human-depleted reads was performed with MetaPhlAn v. 4.0.6 [14] (database version mpa_vOct22_CHOCOPhlAnSGB_202212) with addition of the parameters –ignore_eukaryotes, –ignore_usgbs, and -t rel_ab_w_read_stats.

The taxonomic data was processed with R v. 4.3.0 [15] in RStudio v. 2023.06.0 [16] and ggplot2 v. 3.5.0 [17] was used for visualisations. Beta diversity analysis was performed using QIIME2 v. 2023.09 [18] using the species level estimated read counts generated by MetaPhlAn4. Aitchison distance was used as beta diversity metric to account for the compositionality of the data [19], and was calculated using the QIIME2 diversity plugin adding a pseudocount of 1. Principal-coordinate analysis was calculated using the ecodist R-package v. 2.0.9 [20].

Shannon diversity was calculated using the microbiome package [21]. Differences in Shannon diversity between preservation methods were tested with the Wilcoxon signed rank test.

## Results

### Study participants

The 12 participants delivered 24 faecal samples (Supplementary Table 1). Baseline data with a distribution of the age and sex of the study participants can be found in Supplementary Table 2. The participants with CD had an abnormal level of faecal calprotectin at the time of sample delivery, ranging from 226 to 914 mg/kg, indicating inflammatory activity in the intestine.

### Sequencing reads

Following DNA extraction, the DNA yield was high in all samples, but lower in the samples collected with ethanol compared to those without (mean 144 ng/µL, range and 180 ng/µL respectively). During library preparation, the DNA input was normalised to similar concentrations.

**Fig. 2:**
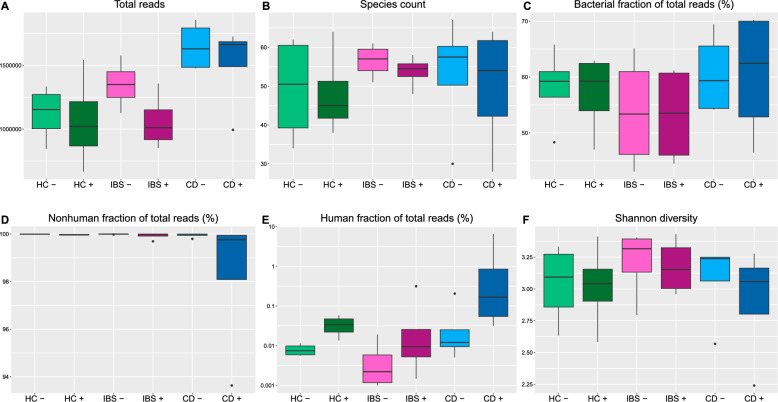
Total reads (**A**), species count (**B**), bacterial fraction of total reads (%) (**C**), nonhuman fraction of total reads (%) (**D**), human fraction of total reads (%) (**E**), and Shannon diversity (**F**) divided into groups of participants: healthy controls (HC), irritable bowel syndrome (IBS), and Crohn’s disease (CD) with no preservative (−) versus 96% ethanol ( +)

Sequencing of the included samples resulted in a median of 1,094,598 quality trimmed sequencing reads (range 666,880-1,726,076) for the samples preserved in ethanol, and a median of 1,371,445 quality trimmed reads for the samples without preservatives (range 844,750-1,854,598) (Fig. 2A). As a result of more total sequencing reads, analyses of faeces without preservatives also resulted in more bacterial reads and a higher species count compared to faeces preserved in ethanol, while a similar bacterial fraction of total reads was observed (Fig. 2A-C).

### Microbial composition

**Fig. 3:**
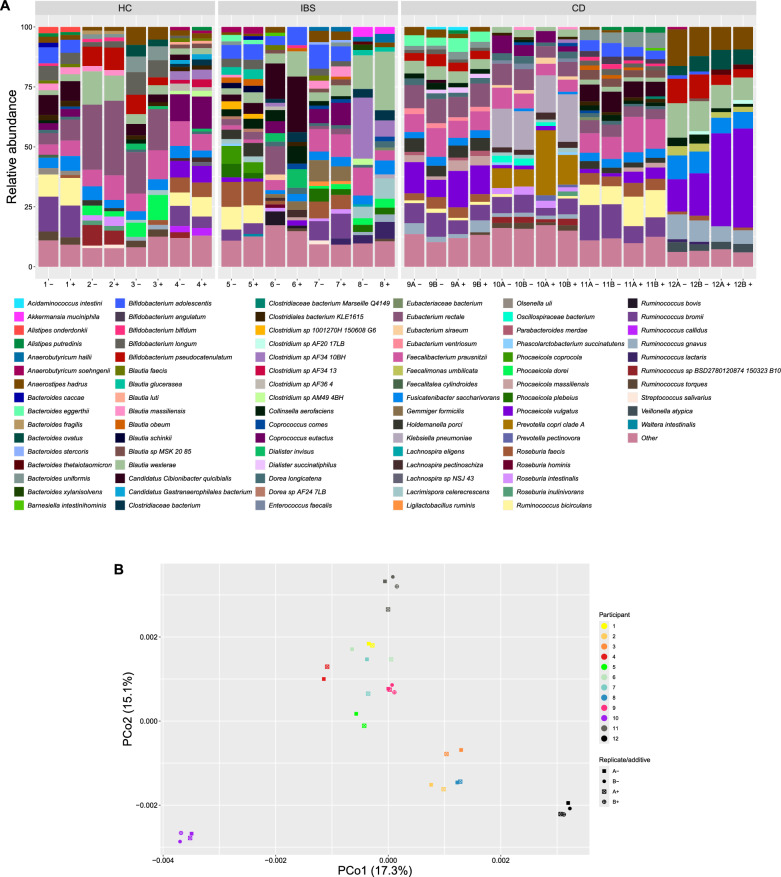
Relative abundance of the bacterial species (**A**) and the beta diversity (**B**) detected in healthy controls (HC) (participant 1–4), irritable bowel syndrome (IBS) (participant 5–8), and Crohn’s disease (CD) (participant 9–12) without a preservative (−) and with 96% ethanol ( +). The two times a CD sample was DNA extracted and sequenced are marked with A and B for the first and second analyses, respectively. Species detected at a relative abundance < 1% are grouped as Other

A total of 197 different bacterial species were identified across the 32 sequenced samples. The identified species belonged to 92 different genera, 37 families, and 10 phyla. Among these, 90 different bacterial species were found with a relative abundance above 1%. Figure 3A provides an overview of the relative abundance of these species with a comparison of (1) the gut microbiome among the different participants, (2) the faecal collection methods ethanol vs. no preservatives, and (3) the first and second time a faecal sample was DNA extracted and sequenced. In general, only small differences were observed between samples collected in tubes with and without the preservative. The variations of the microbiome profiles clustered by each participant and not by groups of participants (i.e., healthy controls, IBS, and CD) or by the type of preservation. For participants number 6 and 7, the variation was slightly more visible (Fig. 3B).

There were no significant differences in Shannon diversity between all samples with or without ethanol (*p* = 0.3) or when divided into groups of participants (healthy controls *p* = 1.0, IBS *p* = 0.9, CD *p* = 0.3).

### Intestinal inflammation and human versus nonhuman reads

We obtained more data from participants with CD than from participants with IBS and healthy controls (median 1,853,583 from CD, 1,341,691 from IBS, and 1,183,483 from healthy controls) (Fig. 2A). However, the fraction of nonhuman reads was similar for all groups except for CD samples collected in tubes with ethanol. As presented in Fig. 2D, the other groups had a nonhuman fraction of total reads above 99.9%, and the human fraction of total reads was correspondingly lower in these groups compared to the CD samples with ethanol (Fig. 2E).

### Sample homogeneity

To assess sample homogeneity, two separate sub-samples were collected for DNA extraction and sequencing from each of the CD samples (Supplementary Table 1). The results showed no major differences between the first and the second time a CD sample was analysed, which demonstrates both the sample homogeneity and the stability of the process from DNA extraction to sequencing (Fig. 3A, B).

## Discussion

In this study, we compared samples collected without preservatives with samples collected in 10 mL of 96% ethanol from patients with GI conditions and healthy controls. To our knowledge, similar GI patient-specific studies with a comparison of collection methods have not previously been performed.

The collection methods in this study differ from the gold standard by samples not being frozen immediately after collection and—as for the ethanol samples—also the addition of a preservative. The time interval of up to 24 h from defecation to freezing at – 80 °C imitates what is practically feasible in most human clinical trials. This particular issue was addressed by Carroll et al. [22] who compared storage temperatures in different time intervals for participants with IBS and healthy controls. The gut microbial composition and diversity of both faecal samples stored at room temperature for 24 h and those preserved at – 80 °C for 6 months exhibited a stronger resemblance to the individual they were collected from than to any other sample [22]. Other studies have shown that the greatest impact on the gut microbiome occurs beyond the 24-h interval [2, 23].

Our main aim was to investigate the differences between the two faecal collection methods regarding suitability for gut microbiome analyses and ease of use. Gut bacterial DNA was successfully analysed in samples both with and without ethanol. However, a slightly higher number of sequencing reads and thus different bacterial species were observed in samples without ethanol. In a systematic review from 2023, Li et al. [2] reported that among eight studies studying the differences between the gold standard and preservation methods for 24-h storage, none of the studies found statistical differences in alpha diversity for any of the different preservation methods compared to the gold standard [2].

We assessed the methods on faeces from both participants with intestinal inflammation (CD + high levels of f-calprotectin), non-inflammatory GI disorder (IBS), and healthy controls. Our hypothesis was, that more human DNA would be detected in samples from participants with CD due to immunological reactions in the intestine. We generally obtained more sequencing reads from CD samples than from IBS and healthy controls, which naturally resulted in more human and non-human data. However, when comparing the human/non-human fraction of total reads, only the CD samples preserved in ethanol (CD +) differed notably from the remaining samples and contained slightly more human DNA. The higher human/lower non-human fraction in these samples does not favour the use of ethanol in this particular study setup.

When considering user-friendliness, we observed a few disadvantages with the ethanol samples. A standardised faecal sample collection method should be simple, intuitive and easy to work with in the laboratory. The risk of spilling and the uncertainty of how well the stool and liquid should be mixed argue against using ethanol. In addition, our laboratory personnel preferred to work with the samples without preservatives, as they were easier to handle due to the sample consistency.

A secondary aim of this study was to evaluate faecal sample homogeneity, which was tested on the CD samples. When studying the microbiome composition, we observed that DNA extraction and sequencing from two different locations in the same stool sample resulted in comparable results. Thus, we also suggest that DNA extraction and sequencing is a stable process.

Our study suggests that faecal sample collection in tubes without ethanol is the better choice when samples are stored for up to 24 h after defecation until freezing. Also, we find that when faeces are collected from patients with IBD, samples without ethanol perform well in the gut microbiome analyses.

## Limitations

The study is limited by the small sample size, as only four participants were included in each group. For validation of our findings, we recommend a larger cohort.

## Supplementary Information

Additional file 1.

## Data Availability

The datasets generated and/or analysed during the current study are not publicly available because they contain identifiable human genome sequences but are available from principal investigator Andreas Munk Petersen on reasonable request. E-mail: andreas.munk.petersen@regionh.dk.

## Abbreviations

GI: Gastrointestinal
IBS: Irritable bowel syndrome
CD: Crohn’s disease
HC: Healthy controls
IBD: Inflammatory bowel disease
