# Supplementary material for: Faecal sample storage without ethanol for up to 24 h followed by freezing performs better than storage with ethanol for shotgun metagenomic microbiome analysis in patients with inflammatory and non-inflammatory intestinal diseases and healthy controls
Source: BMC Res Notes. 2024 Nov 15;17:340. doi: 10.1186/s13104-024-06999-y (PMC11568685; doi:10.1186/s13104-024-06999-y)
Supplement: Supplementary file 1 — Additional file 1. [file 13104_2024_6999_MOESM1_ESM.docx]

**Supplementary tables**

**Supplementary Table 1.** Number of study participants, faecal samples, and analyses.

|  | Samples w/ 96% ethanol | Samples w/o preservatives | Number of times samples were sequenced | Total number of analyses |
| --- | --- | --- | --- | --- |
| Healthy controls | 4 | 4 | 1 | 8 |
| IBS | 4 | 4 | 1 | 8 |
| CD | 4 | 4 | 2 | 16 |
| Total | **12** | **12** |  | **32** |

***Irritable bowel syndrome (IBS), Crohn’s disease (CD)**

**Supplementary Table 2.** Baseline characteristics of the study participants.

|  | Healthy controls | IBS | CD |
| --- | --- | --- | --- |
| n | 4 | 4 | 4 |
| Age, individual (mean) | 1, 4, 35, 41 (20) | 27, 34, 51, 56 (42) | 23, 34, 47, 75 (44) |
| Male, n | 2 | 1 | 3 |
| Female, n | 2 | 3 | 1 |

***Irritable bowel syndrome (IBS), Crohn’s disease (CD)**
